# A different technique in gasless, laparoendoscopic, single-site myomectomy

**DOI:** 10.1007/s00464-020-08044-y

**Published:** 2020-12-14

**Authors:** Guixiu Jin, Xiumin Zhao, Danyang Zhu

**Affiliations:** 1 Department of Gynaecology and Obstetrics, The First People’s Hospital of Taizhou, Hengjie Road 218, Taizhou, 318020 Zhejiang China; 2 Department of Gynecology and Obstetrics, Taixing People′s Hospital, Taixing, Jiangsu China

**Keywords:** Myomectomy, Gasless, Laparoendoscopic, Single-site

## Abstract

**Background:**

The aim of this study was to introduce a novel technique for gasless, laparoendoscopic, single-site (GLESS) myomectomy and to evaluate its feasibility and safety.

**Methods:**

A retrospective observational study was performed at a hospital from Sep 2017 to Nov 2018. 15 patients with symptomatic subserosal or intramural myomas underwent GLESS myomectomy.

**Results:**

The mean age and body mass index were 41.73 ± 8.58 years and 22.72 ± 2.27 kg/m^2^, respectively. 5 patients had a history of abdominal surgery, including four caesarean deliveries and one myomectomy. The mean operative duration, blood loss volume, time to specimen removal, time of bowel activity and postoperative hospitalization duration were 156.47 ± 62.19 min, 57.33 ± 72.35 ml, 29.87 ± 13.6 min, 27.67 ± 10.06 h, and 3.4 ± 0.74 days, respectively. The operation was successful in all patients, there were no surgical or wound complications in any patient, and the histopathological result was leiomyoma in all 15 patients.

**Conclusion:**

The procedure is feasible and safe in selected patients with symptomatic myomas.

**Supplementary Information:**

The online version contains supplementary material available at 10.1007/s00464-020-08044-y.

Uterine myomas are the most frequent benign uterine tumours in reproductive-aged women. Surgery is performed in women with menorrhagia, dysmenorrhea, pelvic pain and enlarged uterine fibroids [1]. With the development of laparoscopic techniques, single-port, laparoscopic myomectomy is currently performed as a minimally invasive surgical technique, which offers the advantages of a more cosmetic result, reduced pain, faster recovery, fewer adhesions and reduced blood loss. However, traditional laparoscopic surgery has numerous adverse effects on cardiopulmonary function due to carbon dioxide pneumoperitoneum [2]. This shortcoming can be avoided using the gasless laparoscopic technique. Gasless laparoscopy can be used in the treatment of benign and malignant gynaecological diseases; in recent years, various surgical instruments and techniques have been used in these procedures [2–6]. The main disadvantages of gasless, laparoendoscopic, single-site (GLESS) surgery are the inline viewing and limited area, which increase the frequency of collisions of laparoscopic instruments extra- and intracorporeally. Therefore, we designed an operation platform to establish a good view in GLESS surgery using an abdominal wall lift system and an umbilical fixation system. This report aims to assess the feasibility and safety of a novel, gasless, single-incision, abdominal access technique for laparoscopic myomectomy.

## Materials and methods

### Clinical data

15 patients who underwent GLESS myomectomy from Sep 2017 to Nov 2018 at The First People's Hospital of Taizhou, Zhejiang, China, were evaluated in this study. A specific informed consent form was signed by all patients, and the study was approved by the institutional review board of The First People's Hospital of Taizhou, Zhejiang, China. All operations were performed by a single surgeon.

The surgical indications were as follows: patients were diagnosed by ultrasonography with symptomatic uterine myoma including menstrual disorders, dysmenorrhea, and infertility or increasing myoma size after follow-up; no other comorbidities were present; uterine preservation was required; fibroid position, size and number were determined by vaginal ultrasound before surgery; cervical or endometrial lesions were excluded. The following demographic characteristics of the patients were collected: age, body mass index (BMI), history of abdominal surgery, location of the myoma, and size and number of myoma(s). The operative duration, estimated blood loss volume, complications, time to specimen removal, pathological results, postoperative hospitalization duration and postoperative visual analogical scale (VAS) pain score were recorded and analysed. The duration of the operation was defined as the period from skin incision to closure, the return of bowel activity as the period from the end of anaesthesia to the first occurrence of bowel gas passage. The degree of postoperative pain was assessed using a VAS at 1 h, 6 h and 24 h postoperatively.

### Surgical access platform and instruments

**Fig. 1:**
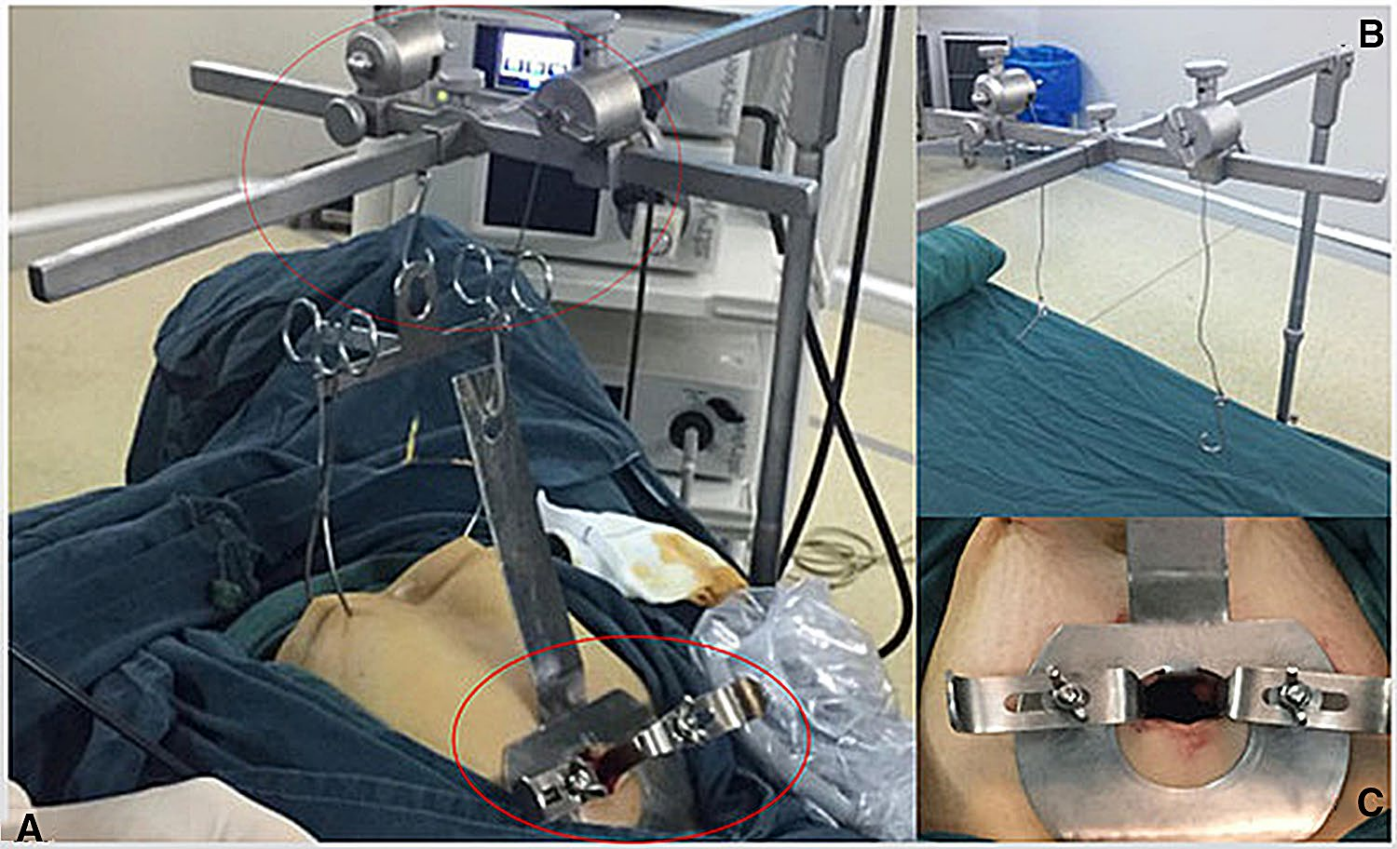
The abdominal wall lifting system and umbilical fixation device. The lifting system (as the red dotted line showed) is composed of a suspension rod, a triangular device and two abdominal wall lifting clamps. The triangular device is used for connecting the tail of the suspension rod and the lifting clamps. The head end of the suspension rod is connected to the umbilical fixation system. The suspension rods are assembled using horizontal and vertical suspension rods to adjust height and width (**B**). The umbilical fixation device (as the red solid line showed) is composed of a stalked perforated steel plate and a hook. The perforated steel plate has a fixator on both sides, which pulls the skin around the umbilicus and serves to fix the umbilicus. The umbilical fixation system can rotate the skin of the umbilicus wound locally in any direction so that the incision plane faces the lesion, and the incision space is completely converted into an operative space, thereby improving the operative efficiency (**C**)

We used an abdominal wall lift system as the method of exposure and a home-made umbilical fixation system as the access device in the GLESS procedure. The abdominal wall lift system (Patent: CN208958194U, CN208958195U) was adopted as the method of exposure in the GLESS procedure (Fig. 1A). The lifting system is composed of a suspension rod, a triangular device and two abdominal wall lifting clamps. The triangular device is used for connecting the tail of the suspension rod and the lifting clamps. The head end of the suspension rod is connected to the umbilical fixation system. The suspension rods are assembled using horizontal and vertical suspension rods to adjust height and width.

The umbilical fixation (Fig. 1C) device is composed of a stalked perforated steel plate and a hook. The perforated steel plate has a fixator on both sides, which pulls the skin around the umbilicus and serves to fix the umbilicus. The umbilical fixation system can rotate the skin of the umbilicus wound locally in any direction so that the incision plane faces the lesion, and the incision space is completely converted into an operative space, thereby improving the operative efficiency.

**Fig. 2:**
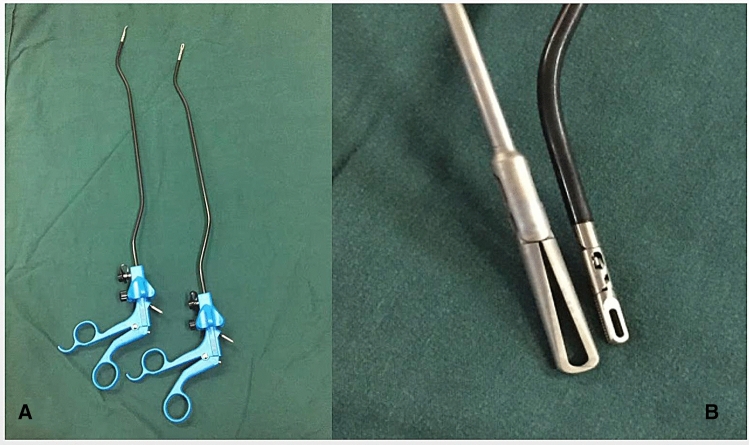
Bent separation forceps (**A**) and myoma-grasping forceps (**B**). Unique bent design for separation forceps to effectively reduce the interference between instruments during the operation (**A**). The myoma-grasping forcep (**B**) shave a thicker head and a thinner handle. Compared with ordinary 5-mm grasping forceps, the designed forcepscan effectively increase the occlusal area and accurately grasp fibroids

Surgical instruments: we designed some special laparoscopic instruments, such as bent separation forceps and myoma-grasping forceps, with a unique bent design to effectively reduce the interference between instruments during the operation. The myoma-grasping forceps have a thicker head and a thinner handle (Fig. 2, Patent: CN208958196U). Compared with ordinary 5-mm grasping forceps, the designed forceps can effectively increase the occlusal area and accurately grasp fibroids.

### Surgical procedure

General anaesthesia was established with the patient in a dorsal lithotomy position on the operating table. A 2–2.5-cm incision was made vertically in the umbilicus to enter the abdominal cavity. Then, the umbilical fixation device was placed in position to be fixed to the abdominal wall, and the lifting system was used to lift the abdominal wall. The lifting system was applied using a cloth towel clamp and lifting the subcutaneous tissue on both sides of the lower abdomen. In this way, the small vertical incision becomes a wider opening and provides an easy orifice for the entry of the laparoscope and the laparoscopic instruments, all of which were reusable.

To reduce haemorrhage in the myoma, 10 IU/100 ml of vasopressin in normal saline was injected into the cervix before myoma removal. All visible myomas were removed. Whilst we used straight instruments, most were custom-designed, bent instruments. The overall procedure for GLESS myomectomy was performed similarly to that for conventional laparoendoscopic single-site surgery (LESS) myomectomy with pneumoperitoneum. However, the extraction of specimens was different from that in the traditional technique. In this study, small myomas were removed directly through the umbilical foramen, whilst large myomas were extracted after being cut into smaller pieces with a knife in the sample bag through the umbilicus, similar to peeling apples; a morcellator was not used.

### Statistical analysis

SPSS version 18.0 (SPSS, Inc., Chicago, IL) was used for statistical analysis. Data are expressed as the mean ± SD (range) for continuous variable and the frequency (percentage) for categorical variables.

## Results

All the procedures were performed successfully using our technique. A uterine manipulator was used in three procedures. The demographic characteristics of the patients are shown in Table 1. The mean age of the patients was 41.27 ± 8.58. The mean BMI was 22.72 ± 2.27 kg/m^2^. 5 patients had a history of previous abdominal surgery: one had a traditional myomectomy, and the remaining four had one caesarean delivery each. The operative findings of the patients are shown in Table 2. The average myoma size 5.79 ± 3.12 cm, and the mean number of myomas removed was 3.87 ± 6.02 (range, 1–25), excluding those smaller than 1 cm. The average blood loss volume was 57.33 ± 72.35 ml. The largest number of myomas was 25, the longest operative duration was 280 min, and the most blood loss volume was 300 ml. None of the patients were treated with blood transfusions. The mean operative duration was 156.47 ± 62.19 min, and the average time of bowel activity was 27.67 ± 10.06 h. The postoperative hospitalization duration was 3.4 ± 0.74 days. The postoperative VAS pain score after 1, 6, and 24 h was 2.07 ± 0.26, 2.07 ± 0.59 and 1.87 ± 1.36, respectively. Three patients used analgesic drugs after the operation.

**Table 1:** Demographic characteristics of the patients

Demographic characteristics	Mean ± SD	Range
Age (years)	41.27 ± 8.58	26–52
BMI (kg/m^2^)	22.72 ± 2.27	19.62–25.96
Abdominal	Caesarean section (4)	
Surgery history	Myomectomy (1)	
Myoma type		
Intramural	3	
Subserosal	2	
Submucosal	0	
Combined	10

**Table 2:** Operative findings of the patients

Clinical outcome	Mean + SD	Range
No. of myomas resected by myomectomy	3.87 ± 6.02	1–25
Size of myoma (cm)	5.79 ± 3.12	1–11
Adhesion		
Yes	3 (20%)	
No	12 (80%)	
Blood loss (ml)	57.33 ± 72.35	20–300
Duration of operation (min)	156.47 ± 62.19	45–280
Removing specimen time (min)	29.87 ± 13.60	12–58
Time of bowel activity (h)	27.67 ± 10.06	17–46
Postoperative hospital time (day)	3.4 ± 0.74	3–5
VAS score for pain		
1st hour	2.07 ± 0.26	2–3
6th hour	2.07 ± 0.59	1–4
24th hour	1.87 ± 1.36	1–5
Analgesic drugs using		
Yes	3 (20%)	
No	12 (80%)	

There were no surgical or wound complications in any patient, and the histopathological result was leiomyoma in all cases.

## Discussion

The present study demonstrates that GLESS myomectomy with our technique is a safe and effective alternative to conventional laparoscopic myomectomy. It is very low in cost compared to the use of trocars in conventional multiport laparoscopy or other gasless, laparoscopic techniques. In addition, the tools are reusable after sterilization and disinfection and could easily be promoted. Since the late 1980s, laparoscopic technology, with a laparoscope, CO_2_ gas and several trocar ports as the main elements, has developed rapidly because of the reduced pain, faster recovery and more cosmetic incision. The desire to develop more minimally invasive surgical techniques has led to a transition from multiple-port surgery to port-less or single-port surgery, known as natural orifice transluminal endoscopic surgery (NOTES) and LESS techniques. In traditional laparoscopic surgery, the pressure of CO_2_ is used to push away the surrounding tissues in the body cavity and provide a satisfactory surgical space. However, CO_2_ can enter the circulatory system through the peritoneum, abdominal organs and broken vessels, resulting in adverse effects on the circulatory system, the respiratory system, and the nervous system, as well as leading to postoperative shoulder pain, nausea and vomiting [7]. Therefore, an increasing number of surgeons are focusing on gasless laparoendoscopic surgery to avoid the detrimental effects of pneumoperitoneum in high-risk patients who have cardiopulmonary or metabolic disorders, and high American Society of Anesthesiologist (ASA) status(≥ II) [8, 9]. In recent years, GLESS has been reported in the treatment of various gynaecologic diseases, including in hysterectomy and adnexal surgery [5, 6, 10–12]. However, GLESS is not widely performed for other gynaecologic conditions because it requires much suturing and knot tying. The major drawback to LESS or GLESS is the frequent collisions of laparoscopic instruments extra- and intracorporeally, ascribed to the lack of triangulation and the limited operating area. Advances in technology have led to the development of special access devices and instruments, providing potential solutions to these problems. Using conventional, rigid, straight instruments, it is difficult to roll threads around an instrument with another instrument to tie intracorporeal knots because these instruments are nearly parallel in single-port laparoscopic surgery.

**Fig. 3:**
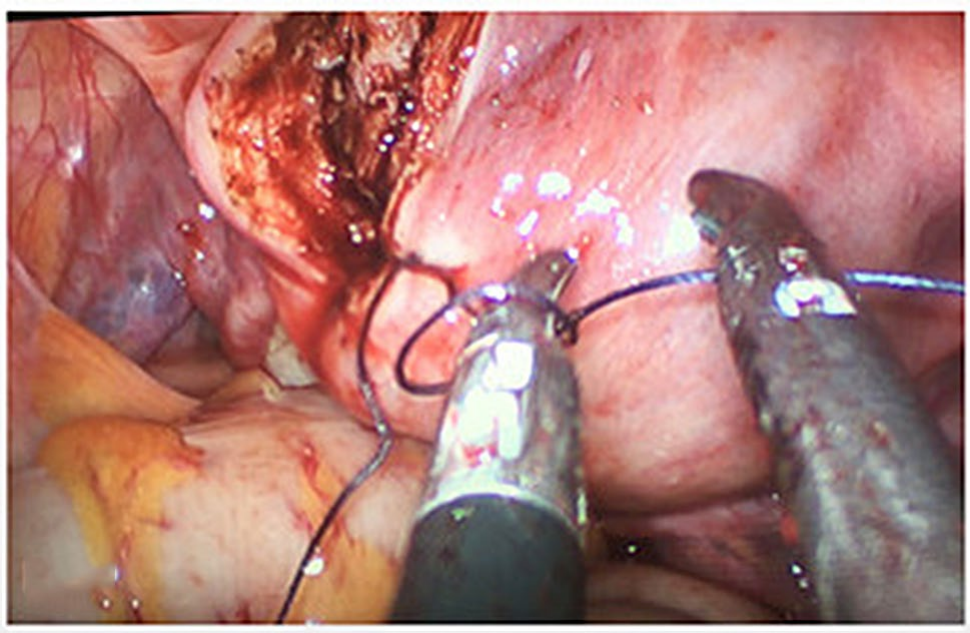
Suturing knot. Suturing knot in the abdominal cavity with no colliding between the instruments

Single-port laparoscopic myomectomy (SP-LM) is difficult to apply because it is inevitably accompanied by technical difficulties, particularly in suturing and knotting. In our study, there was no need to establish a pneumoperitoneum, and a small incision of 2.5 cm was made in the umbilicus to facilitate the suture needle entering, leaving, suturing and knotting which can be performed with a common long-handle needle holder, with a method similar to that applied in conventional laparoscopic surgery (Fig. 3). Choi et al. did continuous suturing tied with a Hem-o-lok clip or suturing by barbed suture to overcome the limitations [13]. By our surgical access platform and instruments, we can easily complete the continuous suturing by pulling the suture extracorporeal which can maintain the tension without instruments collisions, shorten the operation time and reduce bleeding (as shown in the supplementary surgery video). Nearly 30% of patients still need an additional hole to complete the operation as knotting is another difficulty in surgery [14]. Some researchers proposed perform single-port surgery by suturing with robotic surgery, which undoubtedly increase the cost of surgery [15]. Single-port laparoscopically assisted-transumbilical ultraminilaparotomic myomectomy (SPLA-TUM) is another way to reduce the surgery difficult, but it was unsuitable for fibroid which located on the lower posterior wall of the uterus or lateral pelvic wall [16]. In addition, an enlarged umbilical incision may increase the incidence of hernias. Our study showed that GLESS can easily tie a knot extracorporeal, which undoubtedly greatly reduces the difficulty of surgery (as shown in the Online video). We can also easily place gauze though umbilical holder to improve the surgical field of vision which is not possible with traditional gas less laparoscopy.

In our study, we used a gasless, single-port system created with an abdominal wall lifting system and an umbilical fixation system that made transumbilical myoma extraction possible. The umbilical fixation system widens the umbilical incision, and the lifting system completely converts the incision space into the operative space, thereby improving the operative efficiency. In addition, we have designed some special laparoscopic instruments, such as bent separation forceps and myoma-grasping forceps. The unique bent design is useful in preventing crowding and making more room for triangulation.

Although minimally invasive laparoscopic surgery has many advantages when compared to traditional surgery, some difficulty remains in removing large tissue specimens through small incisions. To achieve this, morcellation using either a mechanical power morcellator or a surgical scalpel in the peritoneal cavity should be considered [17]. However, power morcellation-related complications, such as injury to blood vessels and adjacent organs, can occur [18]. This method also carries the risk of unintentional dissemination of the removed tissue in the open peritoneal cavity, which could lead to the recurrence of benign tumours, and even more worrying, the spread of malignant tumours, such as uterine sarcoma, which may cause a decrease in overall survival [19, 20]. In April 2014, the U.S. Food and Drug Administration issued a safety communication warning that power morcellation should not be used due to the risk of intraperitoneal malignant tumour dissemination during laparoscopic surgery [21].

**Fig. 4:**
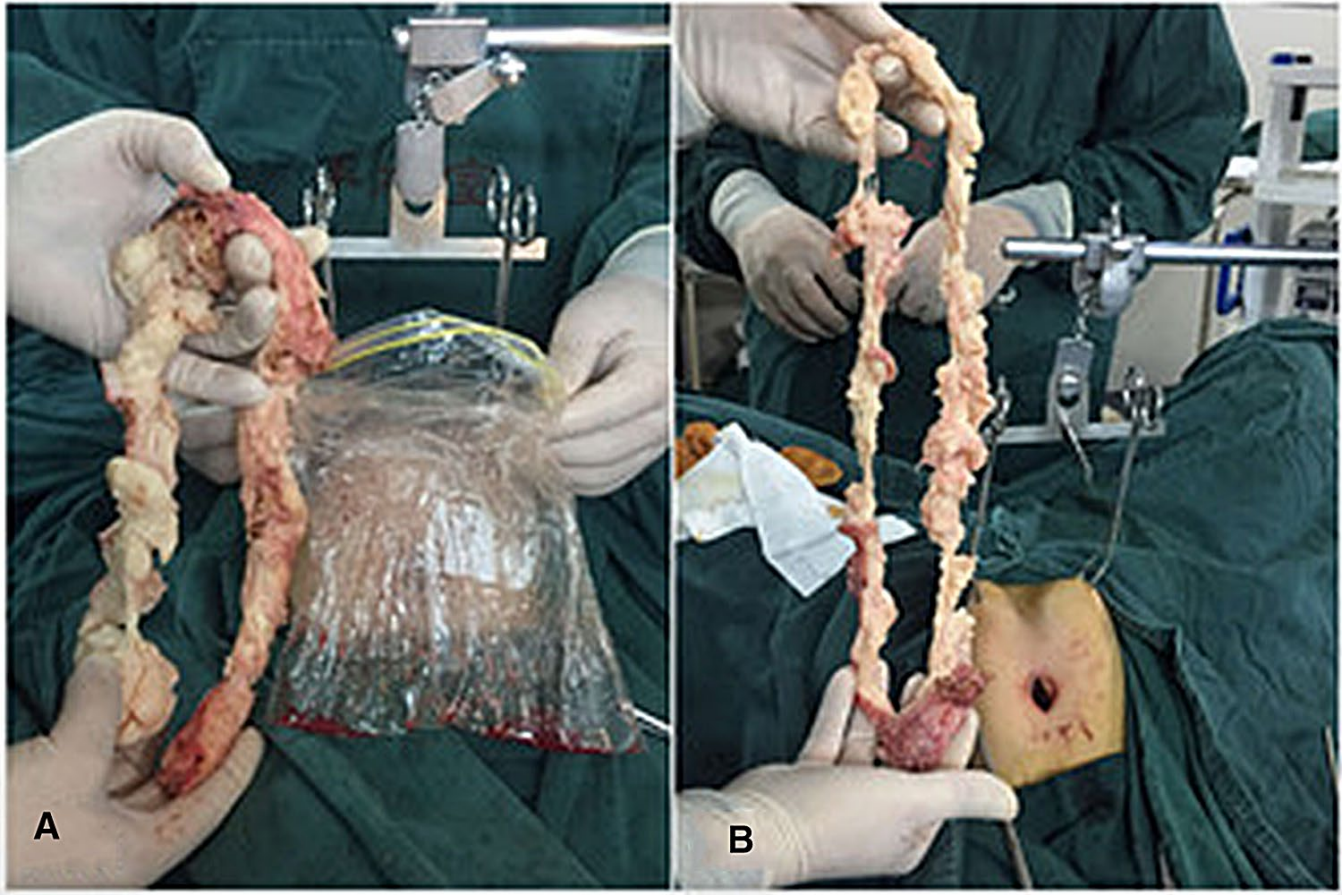
Large myomas removement. Large myomas were put into the sample bag and removed from the umbilical incision by cutting the tissues into small pieces just like peeling an apple

Our homemade, single-port device can be used to easily remove specimens. Small myoma specimens less than 2 cm can be removed directly to avoid omission. Larger myomas can be placed into a sample bag and then removed through the umbilical incision after being cut into small pieces, similar to peeling an apple, to avoid the risk of myoma dissemination under laparoscopy (Fig. 4). In addition, the abdominal lifting system used two lifting clamps to grasp the abdominal wall tissue, avoiding the vascular injury caused by Kirschner wires passing through the subcutaneous tissue of the abdomen.

Although the technique has some advantages, it also has some shortcomings, including a longer operative duration and the use of specialized instruments. Intracorporeal suturing is a significant component of laparoscopic myomectomy and presents an even greater challenge in GLESS surgery; thus, there is a learning curve involved in mastering this technology. Whilst it is difficult to lift the anterior abdominal wall mechanically in obese patients, the abdominal wall can easily be lifted in normal- or low-weight patients. In our study, the patients’ BMI ranged from 19.62 to 25.96. The present study is a pilot study with a small sample and no controls. Prospective controlled studies are needed to determine the safety, advantages and disadvantages of this technique.

## Conclusion

The current study demonstrates that our new technique overcomes many disadvantages of the conventional laparoendoscopic single-site myomectomy technique; it is safe, feasible and inexpensive. However, it represents only the retrospective results of a limited number of cases, and further studies with adequate samples are needed to illustrate its long-term safety.

## Electronic supplementary material

Below is the link to the electronic supplementary material.

Supplementary file1 (MP4 182464 kb)
